# Zishen Qingre Tongluo Formula Improves Renal Fatty Acid Oxidation and Alleviated Fibrosis via the Regulation of the TGF-*β*1/Smad3 Signaling Pathway in Hyperuricemic Nephrology Rats

**DOI:** 10.1155/2021/2793823

**Published:** 2021-12-13

**Authors:** Peng Liu, Chen Wang, Yun Wang, Honghong Zhang, Baoli Liu, Xinping Qiu

**Affiliations:** ^1^Shunyi Hospital, Beijing Traditional Chinese Medicine Hospital, Station East 5, Shunyi District, Beijing 101300, China; ^2^Beijing Hospital of Traditional Chinese Medicine Affiliated to Capital Medical University, 23 Meishuguanhou Street, Dongcheng District, Beijing 100010, China

## Abstract

Hyperuricemia, an independent risk factor for ensuing chronic kidney disease (CKD), contributed to tubulointerstitial fibrosis and insufficiency of renal fatty acid oxidation. Many studies have shown that renal fatty acid oxidation dysfunction is related to the TGF-*β*1/Smad3 signaling pathway. We experimented the effects of Zishen Qingre Tongluo Formula (ZQTF) on the adenine/yeast-induced HN rats and uric acid-induced renal mouse tubular epithelial cells (mTECs), determined whether this effect was related to the TGF-*β*1/Smad3 signaling pathway, and further investigated the relationship between this effect and renal fatty acid oxidation. Rats were given intraperitoneally with adenine (100 mg/kg) and feed chow with 10% yeast for 18 days and then received ZQTF (12.04 g/kg/day) via intragastric gavage for eight weeks. The TGF-*β*1/Smad3 signaling pathway and renal fatty acid oxidation protein were detected by using western blotting, real-time PCR, and immunohistochemistry staining. mTECs induced by UA were used to investigate the relationship between the TGF-*β*1/Smad3 signaling pathway and renal fatty acid oxidation. After treatment with ZQTF, levels of UA, 24 h UTP, BUN, and Scr were significantly decreased and histologic injuries were visibly ameliorated in HN rats. Western blotting, real-time PCR, and immunohistochemistry staining revealed that PGC-1*α*, PPAR*γ*, and PPAR*α* significantly increased, and fibronectin, collagen 1, and P-Smad3 significantly decreased in HN rats and UA-induced mTECs after ZQTF treatment. SIS3 (a specific inhibitor of Smad3) treatment significantly increased the expressions of PGC-1*α*, PPAR*γ*, and PPAR*α* and decreased the expressions of fibronectin, collagen 1, and P-Smad3 in UA-induced mTECs. Our study demonstrated that ZQTF exhibited renoprotective effects by promoting renal fatty acid oxidation via the regulation of the TGF-*β*1/Smad3 signaling pathway. Thus, the present results indicated that ZQTF was a novel antifibrotic strategy for hyperuricemic nephropathy.

## 1. Introduction

Uric acid (UA) is the ultimate product of purine metabolism, and 70% of UA is excreted via the kidneys in the urine [1]. Epidemiological studies show that serum UA is enhanced in patients with chronic kidney disease (CKD) [2]. Considerable evidences highlight that chronic UA injury to the kidney is sufficient to trigger renal tubular injury, tubulointerstitial fibrosis, and glomerulosclerosis, ultimately leading to hyperuricemic nephrology (HN) [3]. Consequently, further exploration of the precise mechanism behind HN progression is urgently required.

Of all these characters, renal interstitial fibrosis, the final common pathway of all progressive CKD, played a central role in HN [4]. Diseased proximal tubules play a critical role in interstitial fibrosis through the release of variety of autocrine and paracrine signals. Although many different types of growth factors and cytokines regulated the renal interstitial fibrosis process, transforming growth factor-*β*1 (TGF-*β*1) is the most potent profibrogenic cytokine [5]. TGF-*β*1 functions by binding to type II TGF-*β* receptors (T*β*RII), transphosphorylation of T*β*RI, and subsequent phosphorylation of Smad2 and Smad3. TGF-*β*1 also could regulate metabolism processes dependently on Smads via the downregulation of peroxisome proliferator-activated receptor *γ* coactivator 1-*α* (PGC-1*α*) protein [6].

PGC-1*α*, one of the key proteins in lipid metabolism, regulates mitochondrial fatty acid metabolism via activating the expression of peroxisome proliferator-activated receptors (PPARs) [7]. PPARs are a family of ligand-activated transcription factors containing three isoforms (PPAR*α*, PPAR*β*/*δ*, and PPAR*γ*). Activated PPAR*α* and PPAR*γ* translocate from the cytoplasm into the nucleus to regulate the genes involved in fatty acid oxidation [8] and transports and disposals to fatty acids [9], respectively. Due to high levels of baseline energy consumption, renal tubular cells preferentially use fatty acids as energy-providing substrates [10]. However, whether UA exposure can influence the fatty acid oxidation in renal tubular cells remains to be seen.

**Figure 1:**
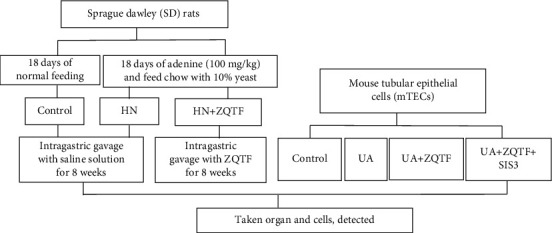
Experimental design.

Zishen Qingre Tongluo Formula (ZQTF), a Chinese herbal medicine, has been used to treat hyperuricemic nephrology. ZQTF was modified from Baihuguizhi Soup, which was from Synopsis of Golden Chamber, and used to treat gout clinically for more than 1800 years ago. However, the role of ZQTF played in the treatment of renal fibrosis in hyperuricemic nephropathy is currently unclear. In this study, the adenine/yeast-induced HN rats were established to study the protective effects of ZQTF on renal fibrosis (Figure 1).

## 2. Materials and Methods

### 2.1. Preparation of ZQTF

ZQTF was a prescriptive granule of traditional Chinese medicine extracted from six natural herbs: Anemarrhena asphodeloides Bunge, Gypsum Fibrosum, Ramulus Cinnamomi, Radix Glycyrrhizae Preparata, coix seed, and Dioscorea nipponica Makino in the ratio of 3 : 8 : 1.5 : 1 : 3 : 5 (*W*/*W*). ZQTF was prepared and standardized at the Yifang Pharmaceutical Co. (Foshan, Guangdong, China). Raw drugs were validated according to the Chinese Pharmacopeia.

### 2.2. Animal Experiments

Eight-week-old male Sprague-Dawley (SD) rats, weighing between 200 and 220 g, were purchased from Beijing Weitonglihua Biotechnology Co., Ltd. Rats were housed under a specific pathogen-free (SPF) environment in temperature- and humidity-controlled incubators at 25°C and 65% relative humidity, with a 12-hour light/dark cycle. Rats had free access to standard rodent chow and tap water ad libitum.

Rats were randomly divided into three groups (each group *n* = 10). The first group and the second group were given intraperitoneally with adenine (100 mg/kg) and feed chow with 10% yeast for 18 days. Then, the first group received ZQTF (12.04 g/kg/day) via intragastric gavage (HN+ZQTF group), while the second group (HN group) and the third group (control group) were administered saline solution. After eight weeks, all rats were euthanized, and the renal tissues, blood, and urine were collected for further analysis.

### 2.3. Serum and Urinary Biochemical Analysis

The rats were housed individually in metabolic cages for 24 h urine collection. 24 h urinary protein (24 h UTP) was measured using an ELISA kit (Exocell, Philadelphia, PA). Blood uric acid (UA), blood urea nitrogen (BUN), and serum creatinine (Scr) were measured on an automated blood biochemistry analyzer (Abbott Diagnostics, Abbott Park, IL, USA). A colorimetric assay kit (Jiancheng, Nanjing, China) quantified the levels of TG in rats and cells, following the manufacturer's protocol.

### 2.4. Renal Tissue Pathology and Immunohistochemistry Staining

The removed kidneys were immediately fixed in a 10% phosphate-buffered formalin solution and then embedded in paraffin. Sections (3 *μ*m thick) were stained with hematoxylin-eosin (HE) and periodic acid-Schiff (PAS) separately to identify kidney structure and were stained with Masson's trichrome to identify the blue collagenous area.

Sections (3 *μ*m thick) were deparaffinized in xylene followed by graded ethanol. Sections were preincubated with sheep serum for 10 min to block nonspecific antigen and then incubated with the corresponding primary antibody (1 : 100) in 5% bovine serum albumin (BSA), overnight at 4°C. After washing with PBS (3 × 5 min), sections were incubated with a biotinylated rabbit antibody for 1 hour, followed by 3 × 5 min washes with PBS. Sections were counterstained with hematoxylin to identify nuclei. Images were quantitatively analyzed using Image-Pro Plus 7.0 software (Media Cybernetics, Bethesda, MD, USA).

### 2.5. Cell Culture and Treatment

Mouse tubular epithelial cells (mTECs) were a gift from Professor HY Lan (Chinese University of Hong Kong) and cultured in DMEM/F12 medium (Life Technologies, Gaithersburg, MD, USA) supplemented with 10% fetal bovine serum (Gibco, Grand Island, NY, USA) at 37°C in a humidified 5% CO_2_ incubator.

Uric acid sodium salt (UA, 1.2 mM) culture medium was made from sodium palmitate (Sigma-Aldrich, Saint Louis, MO, USA) and basal culture medium. The cells were divided into two groups: the control group and the UA group, which were grown with basal culture medium and 1.2 mM UA, respectively. The UA+ZQTF 50 *μ*g/mL, UA+ZQTF 100 *μ*g/mL, and UA+SIS3 groups were treated with 1.2 mM UA+50 *μ*g/mL ZQTF, 1.2 mM UA+100 *μ*g/mL ZQTF, and 1.2 mM UA+0 *μ*M SIS3, respectively.

### 2.6. Cell Viability

The 3-(4,5-dimethylthiazol-2-yl)-2,5-diphenyltetrazolium bromide (MTT) assay was used to determine the effect of ZQTF on cell viability. Exponentially, mTECs growing in 96-well plates were incubated with ZQTF at dosages of 0, 200, 400, 600, 800, and 1000 *μ*g/mL for 24 h. Subsequently, MTT (20 *μ*L, 5 mg/mL) solution was added to each well, and the cells were then incubated for an additional 4 h. Supernatants were then removed, and the formazan crystals were dissolved with 100 *μ*L/well of DMSO and then shaken for 10 min. Optical density (OD) was measured at 490 nm using a microplate reader (BioTek, Winooski, VT, USA).

### 2.7. Western Blotting Analysis

Total protein was extracted from the kidney cortex or isolated mTECs by RIPA lysis buffer containing protease inhibitor cocktail (Juhemei, Beijing, China) and phosphatase inhibitor cocktail (Juhemei, Beijing, China). The following antibodies were used in this study: fibronectin, collagen 1, PGC-1*α*, LXR, PPAR*α*, PPAR*γ*, and *β*-actin (Santa Cruz Biotechnology, Dallas, TX, USA) and P-Smad3 and Smad3 (Cell Signaling Technology, Danvers, MA, USA). Secondary antibodies used were anti-mouse IgG HRP-linked antibodies (Juhemei). Finally, the protein bands were detected by enhanced chemiluminescence (ECL) reagents in the gel imaging system (Thermo Fisher Scientific, Waltham, MA, USA).

### 2.8. Quantitative Real-Time PCR

Total RNA from the kidney cortex and mTECs was extracted using the RNAsimple Total RNA Kit and reverse transcribed to cDNA with by using a RevertAid First Strand cDNA Synthesis Kit (Juhemei). Quantitative real-time PCR was performed and analyzed using a 7500 Fast Real-Time PCR System (Applied Biosystems, Waltham, MA, USA) using UltraSYBR Mixture (Juhemei). The primer sets (Table 1) were used for real-time PCR. All primers for the PCR assay were synthesized by Ruijie (Shanghai, China).

### 2.9. Statistical Analysis

*P* values were calculated by one-way analysis of variance (ANOVA) and independent samples *t*-test to analyze groups of samples in GraphPad Prism 7.0 (GraphPad Software., La Jolla, CA, USA). Values (*P* < 0.05) were considered significant.

## 3. Results

### 3.1. Effects of ZQTF on the General Condition of HN Rats

**Figure 2:**
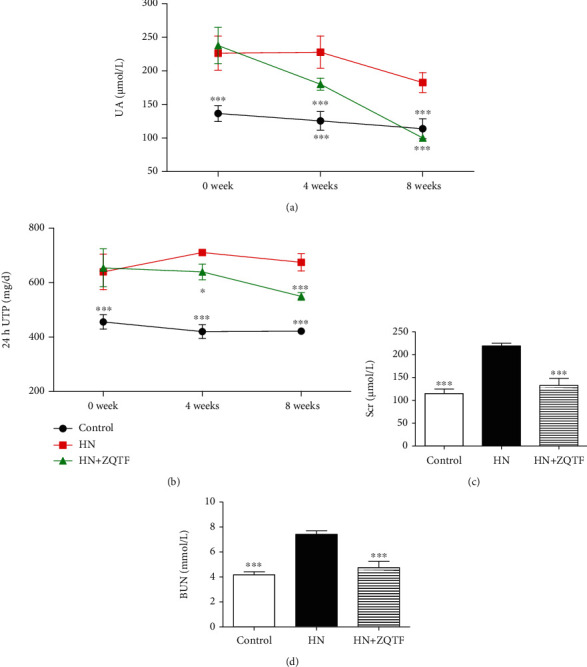
ZQTF attenuated renal injuries in HN rats. (a, b) ZQTF effectively reduced serum UA and 24 h UTP in HN rats. (c, d) ZQTF treated Scr and BUN in HN rats. Data are expressed as mean ± SD (*n* = 10). ^∗^*P* < 0.05 and ^∗∗∗^*P* < 0.001 vs. the HN group.

Compared with the control group, marked increases in UA and 24 h UTP were observed in HN groups (Figures 2(a) and 2(b)). The HN rats showed significant increases in BUN and Scr (Figures 2(c) and 2(d)). After treatment with ZQTF, levels of UA, 24 h UTP, BUN, and Scr were significantly decreased in HN rats compared with levels in the untreated group.

### 3.2. ZQTF Ameliorated Histological Damage and Renal Fibrosis

**Figure 3:**
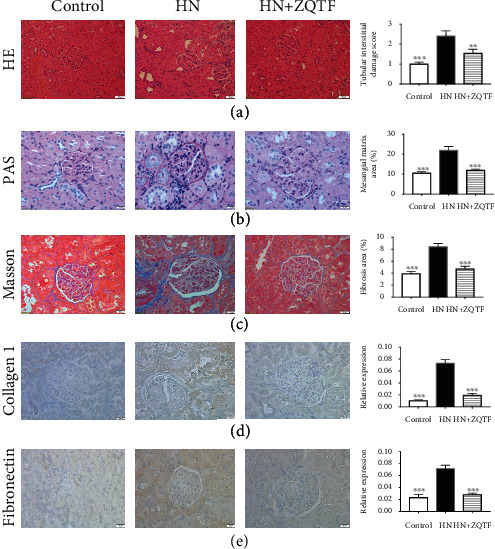
Renoprotective effects of ZQTF on renal tissue pathology. (a–c) HE staining, PAS, and Masson staining (bar = 20 *μ*m). (d) Immunohistochemistry staining of collagen 1 and fibronectin (bar = 20 *μ*m). Data are expressed as mean ± SD (*n* = 10).

HE and PAS staining revealed histologic changes in the kidneys. Kidneys from HN rats showed severe tubulointerstitial injuries with tubular dilatation and swelling (Figures 3(a) and 3(b)). Masson's trichrome staining and immunohistochemistry staining of collagen 1 and fibronectin demonstrated that severe collagen and fibronectin deposition were found in the kidneys of HN rats (Figures 3(c)–3(e)). These histologic injuries were visibly ameliorated by treatment with ZQTF.

### 3.3. ZQTF Ameliorated Renal Fibrosis and Renal Fatty Acid Oxidation

**Figure 4:**
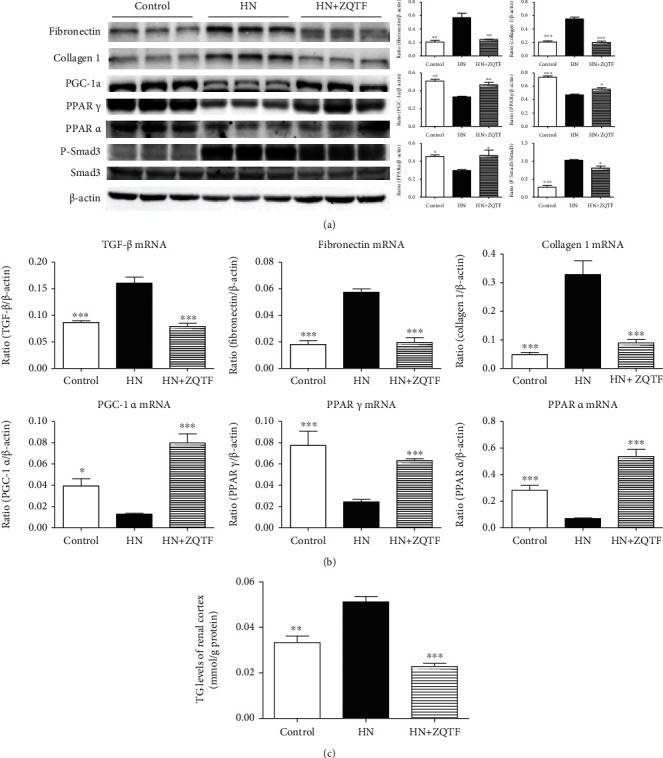
Effects of ZQTF on fibrosis and fatty acid oxidation in renal tissues. (a) Effect of ZQTF on protein levels of fibronectin, collagen 1, PGC-1*α*, PPAR*γ*, PPAR*α*, P-Smad3, and Smad3 by western blot analysis. (b) Effect of ZQTF on mRNA levels of TGF-*β*1, fibronectin, collagen 1, PGC-1*α*, PPAR*γ*, and PPAR*α* by real-time PCR analysis. Data are expressed as mean ± SD (*n* = 10). ^∗^*P* < 0.05, ^∗∗^*P* < 0.01, and ^∗∗∗^*P* < 0.001 vs. the HN group.

Renal fibrosis is highly involved in renal injuries, and fatty acid oxidation plays important roles in providing energy to promote uric acid metabolism. Therefore, we examined the protein and mRNA levels of these factors through western blotting and real-time PCR. Western blotting revealed that PGC-1*α*, PPAR*γ*, and PPAR*α* significantly decreased and fibronectin, collagen 1, and P-Smad3 significantly increased in HN rats, while treatment with ZQTF resulted in a significant protective effect on the kidneys (Figure 4(a)). Results of real-time PCR revealed similar tendencies for TGF-*β*1, fibronectin, collagen 1, PGC-1*α*, PPAR*γ*, and PPAR*α* in the control, HN, and ZQTF-treated groups (Figure 4(b)). In HN rats, quantitative analysis revealed that renal TG levels were highly increased, and ZQTF treatment reduced TG levels (Figure 4(c)).

### 3.4. ZQTF Extenuated Cellular Fibrosis and Promoted Fatty Acid Oxidation in UA-Induced mTECs

**Figure 5:**
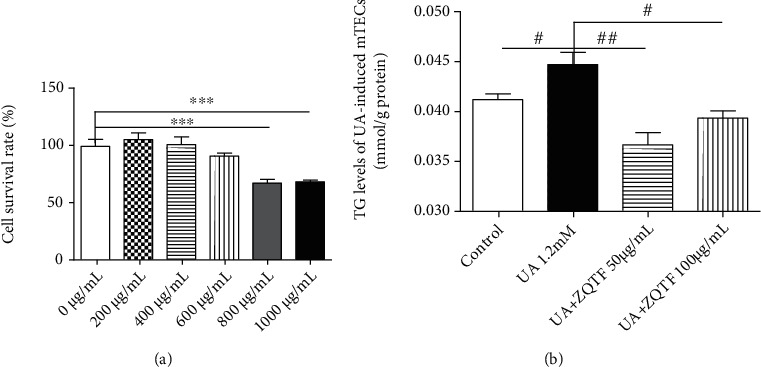
Effects of ZQTF on UA-induced mTECs. (a) Effect of ZQTF on cell viability using MTT assay analysis for 24 h. (b) TG levels in UA-induced mTECs. Data are represented as the mean ± SD for at least 3 independent experiments. ^∗∗∗^*P* < 0.001 vs. the 0 *μ*g/mL group. ^#^*P* < 0.05, ^###^*P* < 0.001 vs. the UA 1.2 mM group.

To examine the most suitable UA dosage, mTECs were cultured in medium supplemented with UA 0, 0.4, 0.8, and 1.2 mM. Due to the most significantly downregulated protein levels of PGC-1*α*, 1.2 mM UA was used to induce the high lipid content cell model (Supplementary Figure 1). We sought to determine the upper limit for ZQTF treatment and used the MTT assay to assess cytotoxicity. A ZQTF concentration over 800 *μ*g/mL showed a cytotoxic effect (Figure 5(a)). Therefore, ZQTF concentrations < 8000 *μ*g/mL were used for subsequent experiments. Quantitative analysis revealed that cellular TG levels were highly increased in the UA-induced mTECs, and treatment with ZQTF 50 and 100 *μ*g/mL decreased cellular TG levels (Figure 5(b)).

**Figure 6:**
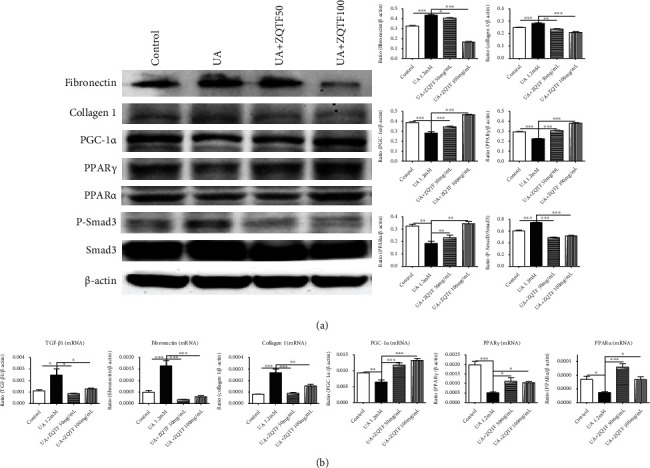
Effects of ZQTF on fibrosis and fatty acid oxidation in UA-induced mTECs. (a) Effect of ZQTF on protein levels of fibronectin, collagen 1, PGC-1*α*, PPAR*γ*, PPAR*α*, P-Smad3, and Smad3 by western blot analysis. (b) Effect of ZQTF on mRNA levels of TGF-*β*1, fibronectin, collagen 1, PGC-1*α*, PPAR*γ*, and PPAR*α* by real-time PCR analysis. Data are represented as mean ± SD for at least 3 independent experiments. ^∗^*P* < 0.05, ^∗∗^*P* < 0.01, and ^∗∗∗^*P* < 0.001 vs. the UA 1.2 mM group.

We then investigated the mechanisms of fatty acid oxidation and cellular fibrosis whereby ZQTF recovered UA-induced mTECs. The protein and/or mRNA expressions of PGC-1*α*, PPAR*γ*, and PPAR*α* significantly decreased, and TGF-*β*1, fibronectin, collagen 1, and P-Smad3 significantly increased in UA-induced mTECs, compared with the control group. Protein and mRNA levels of related markers were reversed in UA-induced mTECs after ZQTF treatment (Figures 6(a) and 6(b)).

### 3.5. ZQTF Extenuated Cellular Fibrosis and Promoted Fatty Acid Oxidation via Suppressing the TGF-*β*1/Smad3 Signaling Pathway by Inhibiting Phosphorylation of Smad3 in UA-Induced mTECs

**Figure 7:**
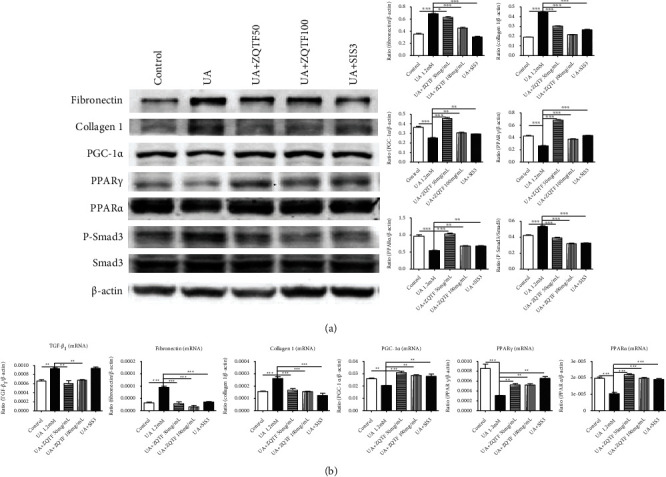
Effects of SIS3 on fibrosis and fatty acid oxidation in UA-induced mTECs. (a) Effect of SIS3 on protein levels of fibronectin, collagen 1, PGC-1*α*, PPAR*γ*, PPAR*α*, P-Smad3, and Smad3 by western blot analysis. (b) Effect of SIS3 on mRNA levels of TGF-*β*1, fibronectin, collagen 1, PGC-1*α*, PPAR*γ*, and PPAR*α* by real-time PCR analysis. Data are represented as mean ± SD for at least 3 independent experiments. ^∗^*P* < 0.05, ^∗∗^*P* < 0.01, and ^∗∗∗^*P* < 0.001 vs. the UA 1.2 mM group.

To better understand how the TGF-*β*1/Smad3 signaling pathway is involved in cellular fatty acid oxidation, we inhibited phosphorylation of Smad3 via treating specific inhibitor of Smad3 (SIS3). We found that SIS3 treatment significantly increased the expressions of PGC-1*α*, PPAR*γ*, and PPAR*α* and decreased the expressions of fibronectin, collagen 1, and P-Smad3 in UA-induced mTECs (Figure 7(a) and 7(b)). As expected, no significant difference in mRNA expressions of TGF-*β*1 was observed with SIS3 treatment (Figure 7(b)). These results suggest that ZQTF may extenuate cellular fibrosis and promote fatty acid oxidation via suppressing the TGF-*β*1/Smad3 signaling pathway by inhibiting phosphorylation of Smad3.

## 4. Discussion

The key finding of this study is that renal fibrosis and the suppression of fatty acid oxidation occurred in HN rats and were associated with activation of the TGF-*β*1/Smad3 signaling pathway. We further found that ZQTF ameliorated hyperuricemic kidney injury by inhibiting renal fibrosis, enhancing renal fatty acid metabolism via downregulating the expression of the TGF-*β*1/Smad3 signaling pathway.

Hyperuricemia is recognized as an independent risk factor for renal damage [11]. Therapies that lower levels of uric acid may retard the progression of renal damage [12]. In the present study, we found that ZQTF dramatically reduced the levels of serum UA in HN rats, which was consistent with the protective effect of ZQTF on renal function. Accumulating evidences indicated that interstitial fibrosis is a major determinant of outcome and a known pathological feature of CKD, including HN [13]. Renal tubular dysfunction and fibrosis of renal tissues induced by soluble UA resulted in HN [14]. Recent evidence suggested that UA is related to the development of renal interstitial fibrosis and is characterized by tubular atrophy and dilatation and increased deposition of the extracellular matrix (ECM) in the renal interstitium [15, 16]. UA might promote epithelial cells for mesenchymal transition in renal tubular cells via decreasing epithelial marker lectins and increasing fibrotic marker *α*-smooth muscle action (*α*-SMA) [17]. TGF-*β*1 is known to be an important cytokine in interstitial fibrosis and glomerulosclerosis via increasing matrix protein synthesis and suppressing matrix degradation in various kidney diseases [5, 18]. In our study, we observed diffuse interstitial fibrosis, upregulation of TGF-*β*1 expression and Smad3 phosphorylation, and collagen I and fibronectin deposition in kidneys of a rat model of HN. Moreover, treatment of ZQTF dramatically alleviated interstitial fibrosis, suppressed TGF-*β* activation and Smad3 phosphorylation, and downregulated expression of collagen I and fibronectin.

Due to fatty acids as energy-providing substrates, fatty acid oxidation plays a core role in maintaining the normal function of renal tubular cells [10]. In HN models, the activation of PPAR*γ* and PPAR*α*, the important markers of fatty acid oxidation, in the kidneys was inhibited, while promoting the expression of PPAR*γ* and PPAR*α* reduced the renal injury [19–21]. Moreover, PPAR*γ* and PPAR*α* reduce kidney damage in many ways. PPAR*γ* activation inhibits hepatic fibrosis by inhibiting the TGF-*β*1/Smad pathway and significantly attenuates unilateral ureteral obstruction-induced renal interstitial fibrosis by reducing the expression of fibronectin, collagen IV, and TGF-*β*1 [13, 22, 23]. Similarly, PPAR*α* activation also inhibits hepatic fibrosis by suppressing Smad2/3 phosphorylation via inhibiting the expression of *α*-SMA and collagen [24]. PPAR*α* activation senses free fatty acids and their derivatives and then translocates from the cytoplasm into the nucleus to regulate the genes involved in fatty acid oxidation [25]. AKI mice with PPAR*α* deficiency have specifically injured proximal convoluted tubules and poor renal function when inflammation increased and fatty acid oxidation reduced [26]. PPAR*α* activation also promotes calcium oxalate-induced renal tubular cell injury [27]. Additionally, PPAR*γ* activation plays a protective role against hyperuricemia-induced inflammatory response and decreases the expression of proinflammatory cytokines and chemokines [28–30]. A PPAR*γ* agonist accelerates glycerol release by increasing fatty acid oxidation [31]. In the present study, we showed that treatment with ZQTF decreased renal and cellular TG levels and promoted fatty acid oxidation via activating the expression of PGC-1*α*, PPAR*α*, and PPAR*γ*.

To explore the mechanisms by which the TGF-*β*1/Smad3 signaling pathway retarded renal fatty acid oxidation, we inhibited phosphorylation of Smad3 via treating SIS3. SIS3 treatment reduced the phosphorylation of Smad3 and ameliorated renal fibrosis by downregulating the expression of fibronectin and collagen I [32]. These data are similar to our own results, whereas previous studies found no evidence that inhibiting phosphorylation of Smad3 could activate the expression of PPAR*α* and PPAR*γ*. More interestingly, our recent study demonstrated that reducing the phosphorylation of Smad3 could upregulate the expression of PPAR*γ* and PPAR*α* via promoting the activation of PGC-1*α*. These findings supported the protection of ZQTF against renal fibrosis in a hyperuricemic nephrology model. One possible explanation is that ZQTF is capable of functioning as an inhibitor of Smad3 and may also promote renal fatty acid oxidation to resist renal damage.

## 5. Conclusion

In conclusion, our study demonstrated that ZQTF exhibited renoprotective effects by the promoting renal fatty acid oxidation via the regulation of the TGF-*β*1/Smad3 signaling pathway. Thus, the present results indicated that ZQTF was a novel antifibrotic strategy for hyperuricemic nephropathy.

## Figures and Tables

**Table 1:** Primer sequences for real-time PCR.

Gene primer sequence (5′-3′)		
Rat		
Collagen 1	F: AGAAAAGGGAACCAAAGG	R: GGAAGCCAGTCATACCAG
Fibronectin	F: GAGGCACAAGGTCCGAGAAGAG	R: GAAACCGTGTAAGGGTCAAAGCA
PGC-1*α*	F: AGACCTGACACAACGCGGACA	R: ATTCTCAAGAGCAGCGAAAGCG
PPAR*α*	F: ATTTGCCAAGGCTATCCCAGG	R: CATCAAGGAGGACAGCATCGTG
PPAR*γ*	F: ACGGTTGATTTCTCCAGCATTTC	R: GGGACGCAGGCTCTACTTTGAT
TGF-*β*1	F: ATTCCTGGCGTTACCTTG	R: CCCTGTATTCCGTCTCCT
*β*-Actin	F: CCGTAAAGACCTCTATGCCAACA	R: CTAGGAGCCAGGGCAGTAATCTC
Mouse		
Collagen 1	F: GAGCGGAGAGTACTGGATCG	R: TACTCGAACGGGAATCCATC
Fibronectin	F: GAGGAGGTCCAAATCGGTCATGTT	R: AACTGTAAGGGCTCTTCGTCAATG
PGC-1*α*	F: CCGAGAATTCATGGAGCAAT	R: TTTCTGTGGGTTTGGTGTGA
PPAR*α*	F: AACATCGAGTGTCGAATATGTGG	R: CCGAATAGTTCGCCGAAAGAA
PPAR*γ*	F: TGCTGAACGTGAAGCCCATCGAGG	R: GTCCTTGTAGATCTCCTGGAGCAG
TGF-*β*1	F: GAGGAGGTCCAAATCGGTCATGTT	R: GCAAGGACCTTGCTGTACTGTGTG
*β*-Actin	F: ACCCTAAGGCCAACCGTGAAAAG	R: CATGAGGTAGTCTGTCAGGT

## Data Availability

The datasets used and/or analyzed during the current study are available from the corresponding author upon request.

## Supplementary Materials

Supplementary Materials Western blot analysis of PGC-1*α* protein in mTECs induced with different concentrations of UA.

## References

[1] Shi Y., Tao M., Ma X. (2020). Delayed treatment with an autophagy inhibitor 3-MA alleviates the progression of hyperuricemic nephropathy. *Cell Death & Disease*.

[2] Li G., Guan C., Xu L. (2020). Scutellarin ameliorates renal injury via increasing CCN1 expression and suppressing NLRP3 inflammasome activation in hyperuricemic mice. *Frontiers in Pharmacology*.

[3] Johnson R. J., Bakris G. L., Borghi C. (2018). Hyperuricemia, acute and chronic kidney disease, hypertension, and cardiovascular disease: report of a scientific workshop organized by the National Kidney Foundation. *American Journal of Kidney Diseases*.

[4] Shi Y., Xu L., Tao M. (2019). Blockade of enhancer of zeste homolog 2 alleviates renal injury associated with hyperuricemia. *American Journal of Physiology. Renal Physiology*.

[5] Gu Y., Liu X., Huang X., Yu X., Lan H. (2020). Diverse role of TGF-*β* in kidney disease. *Frontiers in cell and developmental biology*.

[6] Tiano J. P., Springer D. A., Rane S. G. (2015). SMAD3 Negatively Regulates Serum Irisin and Skeletal Muscle FNDC5 and Peroxisome Proliferator-activated Receptor *γ* Coactivator 1-*α* (PGC-1*α*) during Exercise∗. *The Journal of Biological Chemistry*.

[7] Grabacka M., Pierzchalska M., Dean M., Reiss K. (2016). Regulation of ketone body metabolism and the role of PPAR*α*. *International Journal of Molecular Sciences*.

[8] Bougarne N., Weyers B., Desmet S. J. (2018). Molecular actions of PPAR*α* in lipid metabolism and inflammation. *Endocrine Reviews*.

[9] Marion-Letellier R., Savoye G., Ghosh S. (2016). Fatty acids, eicosanoids and PPAR gamma. *European Journal of Pharmacology*.

[10] Yang X., Okamura D. M., Lu X. (2017). CD36 in chronic kidney disease: novel insights and therapeutic opportunities. *Nature Reviews Nephrology*.

[11] Srivastava A., Kaze A. D., McMullan C. J., Isakova T., Waikar S. S. (2018). Uric acid and the risks of kidney failure and death in individuals with CKD. *American Journal of Kidney Diseases*.

[12] Sampson A. L., Singer R. F., Walters G. D. (2017). Uric acid lowering therapies for preventing or delaying the progression of chronic kidney disease. *Cochrane Database of Systematic Reviews*.

[13] Wang X., Deng J., Xiong C. (2020). Treatment with a PPAR-*γ* agonist protects against hyperuricemic nephropathy in a rat model. *Drug Design, Development and Therapy*.

[14] Pan J., Shi M., Li L. (2019). Pterostilbene, a bioactive component of blueberries, alleviates renal fibrosis in a severe mouse model of hyperuricemic nephropathy. *Biomedicine & Pharmacotherapy*.

[15] Cui D., Liu S., Tang M. (2020). Phloretin ameliorates hyperuricemia-induced chronic renal dysfunction through inhibiting NLRP3 inflammasome and uric acid reabsorption. *Phytomedicine*.

[16] Hügle T., Krenn V. (2016). Histopathophysiology of gout. *Therapeutische Umschau*.

[17] Ryu E. S., Kim M. J., Shin H. S. (2013). Uric acid-induced phenotypic transition of renal tubular cells as a novel mechanism of chronic kidney disease. *American Journal of Physiology. Renal Physiology*.

[18] Isaka Y. (2018). Targeting TGF-*β* signaling in kidney fibrosis. *INTERNATIONAL JOURNAL OF MOLECULAR SCIENCES*.

[19] Hong W., Hu S., Zou J. (2015). Peroxisome proliferator-activated receptor *γ* prevents the production of NOD-like receptor family, pyrin domain containing 3 inflammasome and interleukin 1*β* in HK-2 renal tubular epithelial cells stimulated by monosodium urate crystals. *Molecular Medicine Reports*.

[20] Kanemitsu T., Tsurudome Y., Kusunose N. (2017). Basis of circadian production of uric acid. *Journal of Biological Chemistry*.

[21] Wang J., Zhu X. X., Liu L., Xue Y., Yang X., Zou H. J. (2016). SIRT1 prevents hyperuricemia via the PGC-1*α*/PPAR*γ*-ABCG2 pathway. *Endocrine*.

[22] Choi J. H., Jin S. W., Choi C. Y. (2017). Capsaicin inhibits dimethylnitrosamine-induced hepatic fibrosis by inhibiting the TGF-*β*1/Smad pathway via peroxisome proliferator-activated receptor gamma activation. *Journal of Agricultural and Food Chemistry*.

[23] Jiang L., Chen X. P., Long Y. B. (2015). The potential signaling pathway between peroxisome proliferator-activated receptor gamma and retinoic acid receptor alpha in renal interstitial fibrosis disease. *Journal of Receptor and Signal Transduction Research*.

[24] Chen L., Li L., Chen J. (2015). Oleoylethanolamide, an endogenous PPAR-*α* ligand, attenuates liver fibrosis targeting hepatic stellate cells. *Oncotarget*.

[25] Yao L., Cao B., Cheng Q. (2019). Inhibition of soluble epoxide hydrolase ameliorates hyperhomocysteinemia-induced hepatic steatosis by enhancing *β*-oxidation of fatty acid in mice. *American journal of physiology. Gastrointestinal and liver physiology*.

[26] Iwaki T., Bennion B. G., Stenson E. K. (2019). PPAR*α* contributes to protection against metabolic and inflammatory derangements associated with acute kidney injury in experimental sepsis. *Physiological Reports*.

[27] Su B., Han H., Ji C. (2020). MiR-21 promotes calcium oxalate-induced renal tubular cell injury by targeting PPARA. *American Journal of Physiology. Renal Physiology*.

[28] Cho R. L., Yang C. C., Tseng H. C., Hsiao L. D., Lin C. C., Yang C. M. (2018). Haem oxygenase-1 up-regulation by rosiglitazone via ROS-dependent Nrf2-antioxidant response elements axis or PPAR*γ* attenuates LPS-mediated lung inflammation. *British Journal of Pharmacology*.

[29] Deng J., Xia Y., Zhou Q. (2019). Protective effect of rosiglitazone on chronic renal allograft dysfunction in rats. *Transplant Immunology*.

[30] Shi M., Guo F., Liao D. (2020). Pharmacological inhibition of fatty acid-binding protein 4 alleviated kidney inflammation and fibrosis in hyperuricemic nephropathy. *European Journal of Pharmacology*.

[31] Sergio C. R., Rossana C. Z., Mónica F. M., Isela S. R., Omar A. H. (2020). Lugol increases lipolysis through upregulation of PPAR-gamma and downregulation of C/EBP-alpha in mature 3T3-L1 adipocytes. *Journal of Nutrition and Metabolism*.

[32] Zhang Y., Meng X. M., Huang X. R., Lan H. Y. (2018). The preventive and therapeutic implication for renal fibrosis by targetting TGF-*β*/Smad3 signaling. *Clinical Science (London, England)*.

