# Supplementary figures and images for: Zishen Qingre Tongluo Formula Improves Renal Fatty Acid Oxidation and Alleviated Fibrosis via the Regulation of the TGF-β1/Smad3 Signaling Pathway in Hyperuricemic Nephrology Rats
Source: Biomed Res Int. 2021 Dec 13;2021:2793823. doi: 10.1155/2021/2793823 (PMC8687854; doi:10.1155/2021/2793823)

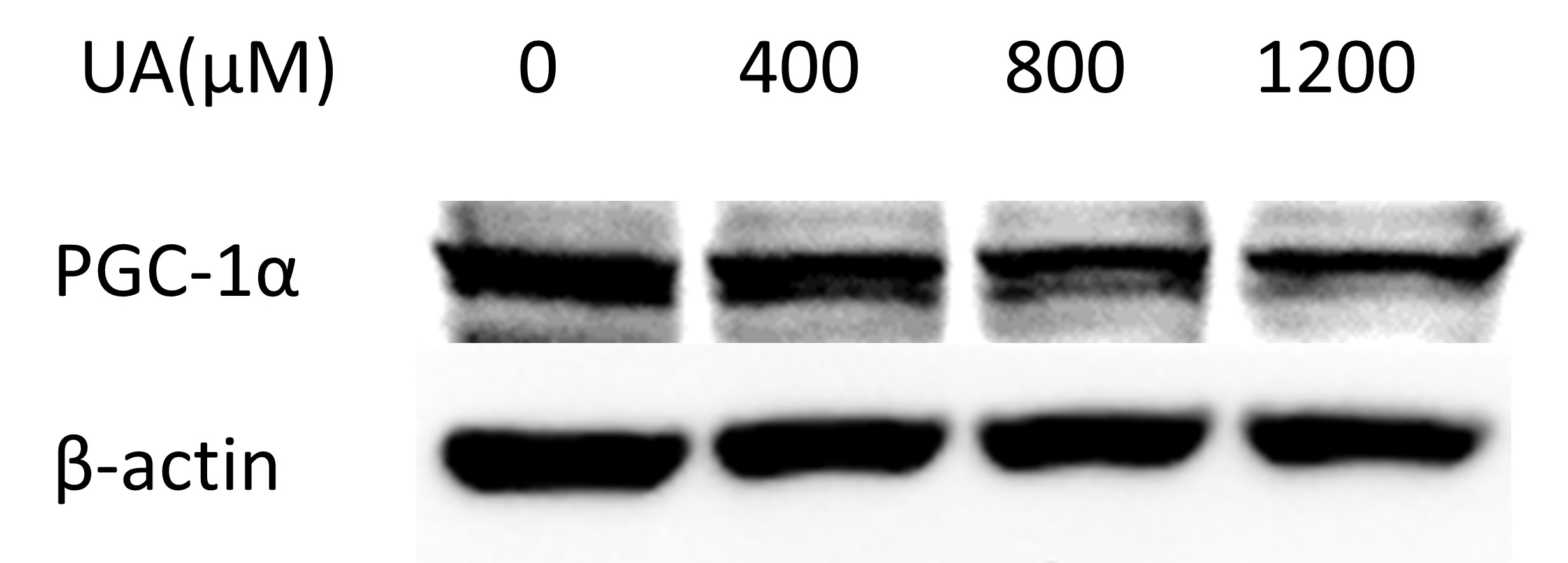

Supplement: Supplementary Materials — Western blot analysis of PGC-1α protein in mTECs induced with different concentrations of UA. [file 2793823.f1.jpg]
